# The proper correction of the mechanical axis in high tibial osteotomy with concomitant cartilage procedures*—*a retrospective comparative study

**DOI:** 10.1186/s13018-019-1333-4

**Published:** 2019-08-28

**Authors:** Myung Ku Kim, Bong Sung Ko, Joo Hyun Park

**Affiliations:** Department of Orthopedic Surgery, College of Medicine, Inha University Hospital, 7-206, 3-Ga Sinheung-dong, Jung-gu, Incheon, 400-711 Republic of Korea

**Keywords:** High tibial osteotomy (HTO), Mechanical axis, Mechanical axis deviation, Cartilage procedures

## Abstract

**Background:**

The guidelines to correct the mechanical axis in high tibial osteotomy (HTO) have changed recently, and some studies have suggested that the correction of the mechanical axis should be based on the severity of cartilage defect. The purpose of this study was (1) to evaluate the radiographic and clinical outcomes of HTO with concomitant cartilage procedures and (2) to compare our method with conventional method regarding the mechanical axis correction.

**Methods:**

Sixty-six knees which underwent opening wedge HTO with cartilage procedures were evaluated retrospectively. The mean age was 56.0 ± 8.3 years, and the average follow-up period was 35.9 ± 22.0 months (range, 24–93 months) with a minimum follow-up of 2 years. All patients were divided into two groups regarding the method of mechanical axis correction; the postoperative mechanical axis was shifted to 50–55% of the tibial plateau width in group I (*n* = 46) and to 62–66% according to the conventional method in group II (*n* = 20). Concomitant cartilage procedures were performed, and each technique of those was determined according to the cartilage status.

**Results:**

The functional scores and visual analog scale for pain in all patients showed a significant improvement at the final follow-up, but there was no significant difference between two groups. The postoperative mechanical axis was the valgus axis of 0.7° in group I with average mechanical axis deviation (MAD) of 51.7%, whereas the valgus axis of 4.2° in group II with average MAD of 64.0%. In patients who underwent second-look arthroscopy, the cartilaginous regeneration could be obtained by cartilage procedures.

**Conclusion:**

In HTO with concomitant cartilage procedures, the method to correct postoperative mechanical axis to the neutral or valgus axis less than 3° could be an effective and safe method to obtain reliable clinical outcomes without complications. Therefore, our method can be used as a selective technique to prevent complications related to the postoperative valgus alignment in patients who are needed much correction angle due to the preoperative severe varus alignment.

**Study design:**

Retrospective comparative study, level III.

## Introduction

High tibial osteotomy (HTO) has been established as an effective surgical intervention for relatively young patients with unicompartmental osteoarthritis of the knee. Through the development of a locking plate design to provide more stability, the medial opening wedge osteotomy has recently become a more common procedure [1]. The most widely used and conventional method to correct mechanical axis for HTO, which was first suggested by Dugdale et al. [2], is a lateral shift of the weight-bearing line (WBL) to 62–66% of the width of the plateau. This point—called the Fujisawa point—matches over the mechanical axis with 3–5° valgus and is laterally located to the lateral tibial spine [3].

The use of cartilage procedures has further expanded for young patients with knee pain and joint surface defects, such as microfracture, the autologous matrix-induced chondrogenesis (AMIC) procedure, autologous chondrocyte implantation (ACI), or stem cell implantation [4–7]. These procedures can play a role in promoting cartilaginous regeneration and improving cartilage status on the weight-bearing area. There is likely a synergistic relation between cartilage procedures and knee realignment, with improved cartilage status and pain relief after HTO [8, 9]. Because cartilage defects can be somewhat normalized through these procedures, we assumed that conventional method to induce postoperative 3–5° valgus axis cannot be mandatory. The guidelines to correct the mechanical axis have changed recently, and some studies have concluded that the correction of the mechanical axis should be based on the severity of cartilage defect [10, 11]. The purpose of this study was (1) to evaluate the radiographic and clinical outcomes of HTO with concomitant cartilage procedures and (2) to compare our method with conventional method regarding the mechanical axis correction.

## Materials and methods

### Study population

**Fig. 1:**
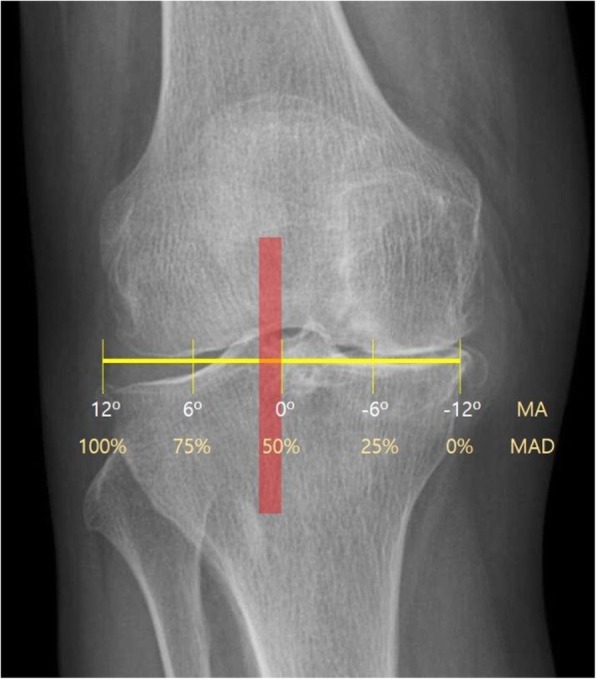
When the target range of postoperative mechanical axis in group I would be determined, it was applied to make a HKA angle of 180–182°. The mechanical axis was shifted to 50–55% of the tibial plateau width near the center of knee joint (shadow box)

**Fig. 2:**
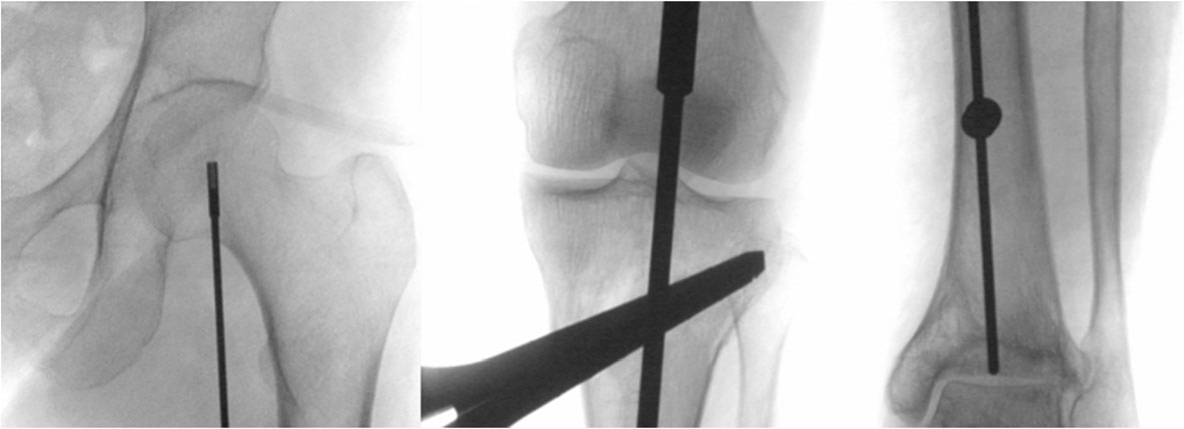
The mechanical axis could be corrected on the intraoperative C-arm using the alignment rod, passing through the midpoint of the medial and lateral tibial spine of the knee joint in group I

**Fig. 3:**
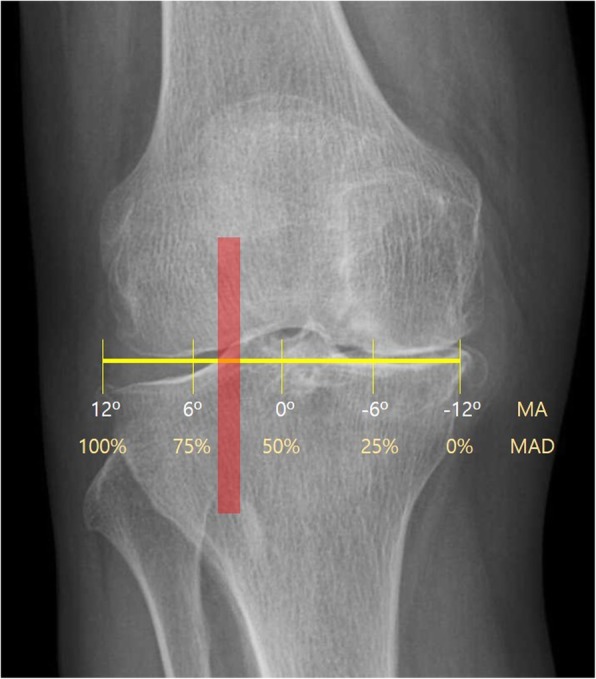
When the target range of postoperative mechanical axis in group II would be determined, it was applied to make a HKA angle of 183–185°. The mechanical axis was shifted to 62–66% of the tibial plateau width, lateral to the lateral tibial spine (shadow box)

The medical records of 66 consecutive patients who had undergone medial opening wedge HTO with concomitant cartilage procedures between April 2010 and July 2016 were reviewed, with a minimum follow-up of 2 years. The patients consisted of 18 men and 48 women, and the mean age of the patients at the time of surgery was 56.0 ± 8.3 years (range, 31–63 years). The average follow-up period was 35.9 ± 22.0 months (range, 24–93 months). All patients were divided into two groups regarding the method of mechanical axis correction; the postoperative mechanical axis was shifted to 50–55% of the tibial plateau width in group I (*n* = 46), and to 62–66% of the plateau width according to the conventional method in group II (*n* = 20). The postoperative mechanical axis was expected to be either neutral or with a valgus axis less than 3° in group I, whereas 3–5° valgus axis according to the conventional method in group II (Figs. 1, 2, and 3). WBL was also measured following the Miniaci guidelines through digital planning such as picture archiving and communication system (PACS) software. There were no statistical differences in the patient’s age, sex, and follow-up period between the two groups.

The inclusion criteria were as follows: (1) patients under the age of 65 had a preoperative magnetic resonance imaging (MRI) for evaluation of cartilage defects, (2) patients underwent medial opening wedge HTO using locking compression plate with concomitant cartilage procedures for treatment of varus alignment, and (3) patients to be available for clinical outcome assessment both preoperatively and for a minimum of 2 years after surgery. Patients who had a history of fractures, systemic inflammatory diseases, such as rheumatoid arthritis, or underwent HTO without any cartilage procedures were excluded. The study protocol was approved by the institutional review board of the senior author’s hospital (INHAUH, 2016-12-003).

### Indications for surgical treatment

Patients under the age of 65 who experienced localized pain in the medial compartment, but wanted to maintain an active lifestyle were also indicated for HTO. Other patient selection criteria included (1) an intact lateral joint compartment prior to surgery, (2) varus deformity more than 3° and less than 20°, (3) flexion contracture ≤ 15°, (4) active range of motion ≥ 90°, (5) absence of moderate-to-severe patellofemoral osteoarthritis, (6) body mass index ≤ 30 kg/m^2^, and (7) failed conservative treatment for a minimum of 6 months with nonsteroidal anti-inflammatory drugs and physical therapy.

### Preoperative radiographic evaluation

Radiography was used to assess the extent of medial osteoarthritis and varus deformity of the knee. The radiological documentation included two radiograph views, comprised of both the bilateral anteroposterior view and bilateral standing posteroanterior view at 45° to obtain a weight-bearing image. The anteroposterior standing whole leg radiograph (WLR) was also done, as it was primarily used for the preoperative measurement of the mechanical axis deviation (MAD), expressed as a percentage of the tibial width (0% is a medial edge, 100% is a lateral edge) and the hip-knee-ankle (HKA) angle. The mechanical axis was measured as the HKA angle formed by lines drawn from the midpoint between the tibial spines to the center of the femoral head proximally and to the center of the talocrural joint distally. Where 180° was equivalent to a straight line, angles greater than 180° indicated a valgus knee position, and angles less than 180° indicated varus alignment. The tibial slope was measured on the lateral view of the tibial shaft. A tangent was marked in Cedara between anterior and posterior borders of the tibial plateau. The angle between this line and the anatomical axis of the tibia minus 90° was determined as a tibial posterior slope [12]. The MRI evaluation was carried out to measure the size of the cartilage defect and check other injuries such as meniscus and ligament tears.

### Surgical intervention

In order to confirm an intact lateral compartment and solve other intra-articular pathologic lesions, the diagnostic arthroscopy was performed first. In case of a symptomatic medial meniscus tear, we performed a partial meniscectomy. Concomitant cartilage procedures were performed, and each technique of those was determined according to the severity of the cartilage defect on arthroscopic findings; if the defect size was ≤ 2 cm^2^, microfracture was done through arthroscopy, and if the defect size was > 2 cm^2^, ACI or stem cell implantation (bone marrow aspirate concentrate or medicinal product composed of allogeneic human umbilical cord blood-derived mesenchymal stem cells) was done after HTO with a mini-open technique.

A 6 cm-sized vertical incision was made over the center of the knee between the medial aspect of the tibial tuberosity and the posteromedial aspect of the tibia, just below the joint line. From the medial border of the patellar tendon, subperiosteal dissection was carried out towards the posteromedial aspect of the tibia, taking care to preserve the distal insertion of the superficial medial collateral ligament, and a blunt Hohmann retractor was inserted to protect the neurovascular structures. Two guide pins were inserted at a point between 3.5 and 4.0 cm below the medial joint line and passed obliquely 1 cm below the lateral articular margin of the tibia towards the tip of the fibular head. The osteotomy was performed in an L-shape following the biplane method. The first osteotomy was performed distal to the K-wire, parallel to the tibial slope. The second frontal osteotomy plane started in the anterior one-third of the proximal tibia at an angle of 110° to the first osteotomy plane. This osteotomy exited the bone proximal to the insertion of the patellar tendon. The osteotomies were performed with an oscillating saw and were completed with chisels. The osteotomy was opened by stepwise insertion of three chisels to avoid intra-articular fractures of the tibial plateau. The mechanical axis was then adjusted according to the preoperative planning strategy and the correction retained with a bone spreader that was inserted into the posteromedial osteotomy gap. The locking compression plate (OhtoFix®, Ohtomedical Co. Ltd., Goyang, South Korea) was inserted into the subcutaneous tunnel and then centered on the anteromedial plane of the tibia. The proximal fixation of the plate was carried out with three locking head screws in the subcortical area. The plate was then pretensioned by inserting a temporary lag screw distal to the osteotomy. For definitive fixation of the plate, the distal locking head screws were inserted through a small incision. If needed, the osteotomy gap was closed with allobone graft. A natural drainage was inserted but placed away from the osteotomy gap.

### Postoperative management

In patients with a small-sized cartilage defect restored by microfracture, the range of motion exercises and partial weight-bearing using crutches were allowed at 5 days postoperatively, and full weight bearing was permitted at 6 weeks after surgery. However, in patients with a medium-to-large-sized cartilage defect restored by either ACI or stem cell implantation, partial weight-bearing with crutches was allowed at 8 weeks postoperatively, and full weight-bearing was permitted at 12 weeks after surgery. The plate removal was usually recommended to patients after one and a half years postoperatively, and the cartilaginous regeneration could be confirmed on the weight-bearing area at that time.

### Assessment of radiographic and clinical outcomes and arthroscopic evaluation of cartilaginous regeneration

The anteroposterior standing WLR was included in postoperative radiographic follow-up in order to evaluate the postoperative alignment of the knee joint through evaluation of MAD, HKA angle, posterior tibial slope, and evaluating for any complications. Clinical outcomes were compared preoperatively and at the final follow-up by the Knee injury and Osteoarthritis Outcome Score (KOOS), subjective form of International Knee Documentation Committee (IKDC) score, and visual analog scale for pain (pVAS). The cartilage status on arthroscopic findings was confirmed in patients who were able to have a second-look arthroscopy at the time of hardware removal, and the degree of cartilage regeneration was estimated through the International Cartilage Repair Society (ICRS) macroscopic cartilage evaluation score [13].

### Statistical analysis

The metrics of two groups regard to compare pVAS, functional scores, HKA angle and MAD were evaluated for normality using the Shapiro-Wilk test. Statistical differences of metrics with normal distributions were evaluated using the independent and paired *t* test for data. Non-parametric analysis (Mann-Whitney *U* and Wilcoxon signed-rank test) was used to compare data found not to be normal distributions. The chi-square test (*χ*^*2*^) was used to analyze categorical variables in order to compare patients’ sex. The level of statistical significance was set to *P* < 0.05. All statistical analyses were performed using the IBM Statistical Package for the Social Sciences (SPSS) software version 19.0 (Chicago, IL, USA).

## Results

The average pVAS in all patients improved from 7.1 ± 1.7 points preoperatively to 1.0 ± 0.8 points at the final follow-up. The functional scores, KOOS and IKDC score, also showed a significant improvement after surgery (66.2 ± 18.5 to 105.0 ± 12.4 and 27.2 ± 13.0 to 68.0 ± 6.7, respectively; all *P* < 0.001). However, there was no significant difference in functional scores and pVAS between the two groups at the final follow-up (Table 1).

**Table 1:** Clinical and radiographic outcomes of patients between two groups

Variable	Period	Group I (*n* = 46)	Group II (*n* = 20)	*P* value
KOOS (point)	Preop	66.5 ± 19.7	65.6 ± 15.8	0.846
	Final f/u	105.9 ± 12.7	102.9 ± 11.7	0.368
IKDC score (point)	Preop	25.2 ± 10.0	31.9 ± 17.6	0.127
	Final f/u	67.5 ± 6.4	68.9 ± 7.5	0.452
pVAS (point)	Preop	7.2 ± 1.8	6.9 ± 1.4	0.552
	Final f/u	1.0 ± 0.8	1.0 ± 0.7	0.834
HKA angle (°)	Preop	173.9 ± 3.1	173.4 ± 3.3	0.605
	Final f/u	180.7 ± 0.8	184.2 ± 1.3	< 0.001
MAD (%)	Preop	19.0 ± 9.5	14.9 ± 11.5	0.138
	Final f/u	51.7 ± 2.2	64.0 ± 6.7	< 0.001
Posterior tibial slope (°)	Preop	7.9 ± 4.0	8.6 ± 3.4	0.490
	Final f/u	10.4 ± 4.5	10.2 ± 3.6	0.837

The statistical significance was set at *p* < 0.05

*Preop*, preoperative, *F/u* follow-up

The preoperative average HKA angle was 173.8° ± 3.2°, and the mean correction angle of the mechanical axis was 8.0° ± 3.7° (range, 3–22°). The target correction of 180–182° was achieved in group I with an average angle of 180.7° ± 0.8°, and the average angle of 184.2° ± 1.3° was achieved in group II. The average MAD in group I was shifted postoperatively to 51.7% ± 2.2% of the tibial plateau width, compared to 64.0% ± 6.7% in group II (*P* < 0.001). The postoperative posterior tibial slope was 10.4° ± 4.5° in group I, compared to 10.2° ± 3.6° in group II, with no significant differences noted (*P* = 0.837) (Table 1).

**Fig. 4:**
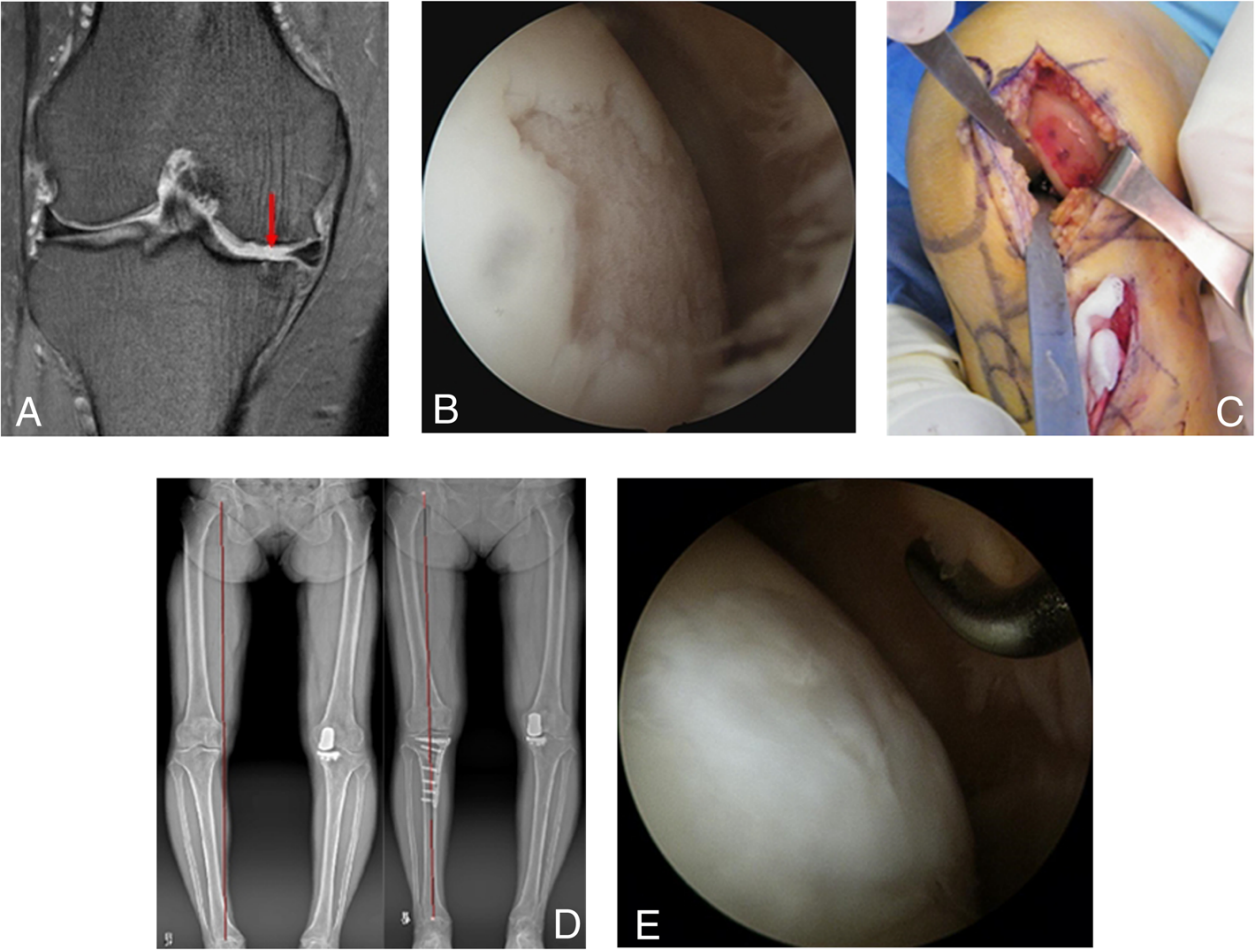
This case was a 59-year-old female suffering from medial osteoarthritis with right knee pain. **a** Large cartilage defect on medial femoral condyle through preoperative MRI. **b** 3.0-cm^2^-sized cartilage defect was confirmed by diagnostic arthroscopy. **c** Concomitant ACI was performed. **d** The mechanical axis could be corrected from 10° varus preoperatively to neutral alignment at the final follow-up. **e** The cartilaginous regeneration (ICRS score, 10 points) could be confirmed at the second-look diagnostic arthroscopy

The average size of the cartilage defect on arthroscopic findings was 2.7 ± 2.2 cm^2^ on the medial femoral condyle, and each patient underwent concomitant cartilage procedures according to the severity of cartilage defect; microfracture in 41 patients, ACI in 14 patients, stem cell implantation (3 patients in bone marrow aspirate concentrate, 8 patients in medicinal product composed of allogeneic human umbilical cord blood-derived mesenchymal stem cells) in 11 patients. A total of 32 patients (48.5%) also underwent second-look arthroscopy at the time of hardware removal, and the cartilaginous regeneration could be obtained by cartilage procedures (Fig. 4). The average ICRS macroscopic cartilage evaluation score of 32 patients improved from 4.0 ± 1.1 points (grade III, abnormal) at the diagnostic arthroscopy to 8.9 ± 0.8 points (grade II, nearly normal) at the second-look arthroscopy (*P* < 0.001).

All patients in the two groups were followed-up until the bony union of the osteotomy site had been radiographically documented. There was no instance of non-union of the osteotomy gap after the tibial osteotomy. No clinical complications such as wound infection (superficial or deep), implant breakage, major loss of correction, or neurovascular injuries were also observed.

## Discussion

The principal finding of this study was that the reliable clinical outcomes were achieved without complications through the opening wedge HTO with concomitant cartilage procedures, which shifted the postoperative mechanical axis to 50–55% of the tibial plateau width. Aiming the midpoint of the medial and lateral tibial spine could have resulted in the postoperative average MAD of 51.7% ± 2.2% with the valgus axis of 0.7° ± 0.8°, and the cartilaginous regeneration could be also obtained by cartilage procedures at the second-look arthroscopy. Therefore, our method can be used as a selective technique to prevent complications related to the postoperative valgus alignment in patients who are needed much correction angle due to the preoperative severe varus alignment.

The aim of a valgus HTO is to transfer the mechanical axis from a position medial to the midline of the knee to a position lateral to the midline of the knee, helping to decrease joint loading and subsequently delay medial joint osteoarthritis. This surgery has already become more popular with relatively young active patients following improvements in surgical technique, fixation devices, and patient selection with fewer complications [14–16]. It may be important to calculate the correction angle through preoperative radiographic images, and then maintain the postoperative mechanical axis continuously. According to Fujisawa et al. [3], the postoperative mechanical axis should pass 1/3 of the distance laterally from the center of the tibial plateau, and Dugdale et al. [2] recommended that postoperative WBL should pass through 62.5% of the width of the plateau. Another study also recommended 3–7° of valgus achieved at operation with significantly better results, and the correction failure may lead to several complications [15].

According to Jakob et al. [10], they also suggested that correction of the mechanical axis should depend on the thickness of the remaining medial compartment cartilage: if 1/3 of medial cartilage is lost, the mechanical axis should pass 10–15% laterally from the center of the tibial plateau; if 2/3 of the cartilage is lost, the axis should pass 20–25% laterally, and if almost all the cartilage is lost, it should pass 30–35% laterally. Kahlenberg et al. [17] concluded that HTO with cartilage procedures provides a reliable improvement in functional status in the medium- to long-term period after surgery and has potential to delay or avoid the need for knee arthroplasty surgery altogether. We usually performed cartilage procedures such as microfracture, ACI, or stem cell implantation related to cartilage defect of the medial femoral condyle, which could promote cartilaginous regeneration on the weight-bearing area. Microfracture is the most reasonable method for small chondral lesions [7], and the AMIC procedure has shown great outcomes in some articles [4]. However, ACI or stem cell implantation can be more reliable on medium-to-large-sized cartilage defect. Oussedik et al. [6] suggested that ACI was more effective than microfracture for lesion more than 4 cm^2^. Kim et al. [5] also concluded that the fibrin-ACI method could be applied for a large chondral lesion with good results, and the fibrin-ACI method is simpler and a less-invasive technique than other ACI. As these various techniques of cartilage procedures could be possible simultaneously for HTO, it would not be absolutely necessary to shift the postoperative mechanical axis to 62.5% of the tibial plateau width as a conventional method.

Valgus HTO may result in latent lateral compartment osteoarthritis. Hernigou et al. [18] concluded that even though the limb should be in valgus alignment postoperatively, too much overcorrection would lead to overloading and rapid deterioration of the lateral compartment. Kwon et al. [19] showed that 53% patients showed progressive degeneration of lateral meniscus after valgus HTO, and Prakash et al. [20] suggested that the increased osteoarthritis on lateral compartment might be due to the result of this increased load on the lateral compartment. The valgus status after HTO can also induce difficult conversion to total knee arthroplasty (TKA) related to ligament balancing. There are many factors and technical difficulties to consider in order to avoid various complications and surgical errors in the conversion of TKA after HTO; most surgeons agree that the skin and soft tissue status, combined deformity, and ligamentous distortion around the knee after previous HTO can make the surgical procedures more difficult [21, 22]. The anatomical deformity and bony distortion of the proximal tibial metaphysis after HTO make the conversion TKA more difficult [21], and Song et al. [23] suggested that as the postoperative change of lower extremity alignment can cause disorder of the collateral ligament and posterior cruciate ligament, causing more difficulty in balancing properly in cases of overcorrected valgus deformity.

In spite of no medial collateral ligament laxity, furthermore, some patients have complained of unsatisfactory cosmetic problems related to the postoperative valgus alignment after conventional HTO in the author’s institution. The authors consider that Asian people have a tendency not to adapt to the postoperative valgus alignment well. In this study, the postoperative mechanical axis was corrected to be either neutral or with a valgus axis less than 3° in group I. Any unsatisfactory cosmetic problem or lateral compartment osteoarthritis related to the postoperative valgus alignment had not occurred until the final follow-up, and we expect to avoid technical difficulties in the case of future TKA conversion. Hence, if the concomitant cartilage procedures would be performed with HTO, it can be appropriate to shift the postoperative mechanical axis to 50–55% of the tibial plateau width compared with conventional method.

There were some limitations in this study. Since our intraoperative method was comprised of non-weight-bearing systems under which the medial compartment space will widen, we could not accurately reflect the weight-bearing body anatomical environment with observation error to the evaluating WLR. Matching the WBL to the target range during osteotomy would usually be difficult, meaning that under- or over-correction of the WBL will be common. Fortunately, the target range of the postoperative mechanical axis could be obtained in all patients without clinical problems related to this difference. Second, it had a limitation regards to the retrospective study of a relatively small number of patients with a minimum follow-up of 2 years. Because no comparative study has analyzed the clinical results related to correct postoperative mechanical axis to the neutral or valgus axis less than 3°, a power study for sample size calculation was not possible, and how patients were divided into two groups could not be presented clearly. However, the follow-up period was sufficient to estimate clinical outcomes or any complications in the investigated cases. Although not a large number in group I, 8 patients showed a symptomatic improvement after surgery without any complications with a follow-up of at least 5 years. Third, although the ICRS score was improved postoperatively in 32 patients through the concomitant cartilage procedures, there was a limitation to determine the effect of cartilage procedures due to the absence of control group with HTO alone. Finally, the concomitant cartilage procedures were performed according to the severity of the cartilage defect on arthroscopic findings, but different types of cartilage procedures were applied between the two groups. Nevertheless, our method to correct the mechanical axis in HTO with concomitant cartilage procedures can propose important clinical implications. If the varus alignment was severe with osteoarthritis preoperatively, much correction angle of the mechanical axis also was required. Although the postoperative mechanical axis is corrected to the neutral or valgus axis less than 3° with concomitant cartilage procedures, reliable clinical outcomes can be achieved through minimal correction without complications such as lateral compartment overloading or unsatisfactory cosmetic problem. Further prospective studies with larger numbers may be also required to follow-up the risk of complications in our method.

## Conclusion

In HTO with concomitant cartilage procedures, the method to correct postoperative mechanical axis to the neutral or valgus axis less than 3° could be an effective and safe method to obtain reliable clinical outcomes without complications. Therefore, our method can be used as a selective technique to prevent complications related to the postoperative valgus alignment in patients who are needed much correction angle due to the preoperative severe varus alignment.

## Data Availability

Not applicable.

## Abbreviations

ACI: Autologous chondrocyte implantation
HKA: Hip-knee-ankle
HTO: High tibial osteotomy
ICRS: International Cartilage Repair Society
IKDC: International Knee Documentation Committee
KOOS: Knee injury and Osteoarthritis Outcome Score
MAD: Mechanical axis deviation
pVAS: Visual analog scale for pain
WBL: Weight bearing line
WLR: Whole leg radiograph
